# Supplementary material for: Change in alcohol consumption and physical activity during the COVID-19 pandemic amongst 76 medical students
Source: PeerJ. 2021 Dec 9;9:e12580. doi: 10.7717/peerj.12580 (PMC8667719; doi:10.7717/peerj.12580)
Supplement: Supplemental Information 2 [file peerj-09-12580-s002.docx]

| Intensity  (Mark all four options) | Amount per week, MET units | | | | |
| --- | --- | --- | --- | --- | --- |
|  | None | <½ hour | 1 hour | 2 – 3 hours | > 4 hours |
| Normal walking or respective | 0 | 69 | 138 | 345 | 550 |
| Brisk walking or respective | 0 | 99 | 198 | 495 | 792 |
| Light jogging or respective | 0 | 210 | 420 | 1050 | 1680 |
| Brisk jogging or respective | 0 | 240 | 480 | 1200 | 1920 |
