# Supplementary material for: Change in alcohol consumption and physical activity during the COVID-19 pandemic amongst 76 medical students
Source: PeerJ. 2021 Dec 9;9:e12580. doi: 10.7717/peerj.12580 (PMC8667719; doi:10.7717/peerj.12580)
Supplement: Supplemental Information 3 [file peerj-09-12580-s003.pdf]

Ikä

Sukupuoli

- Mies
- Nainen

Asutko?

- Yksin
- Jonkun kanssa

Kuuluuko talouteesi koira?

- Kyllä
- Ei

Onko sinulla alle 18-vuotiaita lapsia?

- Kyllä
- Ei

Oletko kilpaurheilija?

- Kyllä
- Ei

Kuinka paljon olet keskimäärin liikkunut viikossa ennen korona-aikaa? (ennen maaliskuu 2020)

	Ei lainkaan	Alle 30 min	Noin tunti	2-3 tuntia	4 tuntia tai enemmän
Kävelyä vastaavaa liikuntaa					
Reipasta kävelyä vastaavaa liikuntaa					
Kevyttä hölkkää vastaavaa liikuntaa					
Reipasta juoksua vastaavaa liikuntaa					

Kuinka paljon olet keskimäärin liikkunut viikossa korona-aikana? (maaliskuu 2020 jälkeen)

	Ei lainkaan	Alle 30 min	Noin tunti	2-3 tuntia	4 tuntia tai enemmän

Kävelyä vastaavaa liikuntaa					
Reipasta kävelyä vastaavaa liikuntaa					
Kevyttä hölkkää vastaavaa liikuntaa					
Reipasta juoksua vastaavaa liikuntaa					

Onko sinulle korona-aikana (maaliskuu 2020 jälkeen) sattunut vammoja tai sairastumisia, jotka ovat vaikuttaneet liikunnan määrään?

- Kyllä
- Ei

Alkoholinkäyttö ennen korona aikaa (maaliskuu 2020)

Kuinka monta kertaa viikossa olet keskimäärin käyttänyt alkoholia ennen korona-aikaa?

Kuinka monta alkoholiannosta kerralla olet keskimäärin käyttänyt ennen korona-aikaa? (yksi alkoholiannos on esimerkiksi pullo keskiolutta, 12 cl viiniä tai 4 cl väkeviä)

Alkoholinkäyttö korona-aikana (maaliskuu 2020 jälkeen)

Kuinka monta kertaa viikossa olet keskimäärin käyttänyt alkoholia korona-aikana ?

Kuinka monta alkoholiannosta kerralla olet keskimäärin käyttänyt korona-aikana? (yksi alkoholiannos on esimerkiksi pullo keskiolutta, 12 cl viiniä tai 4 cl väkeviä)
