# Supplementary material for: Change in alcohol consumption and physical activity during the COVID-19 pandemic amongst 76 medical students
Source: PeerJ. 2021 Dec 9;9:e12580. doi: 10.7717/peerj.12580 (PMC8667719; doi:10.7717/peerj.12580)
Supplement: Supplemental Information 4 [file peerj-09-12580-s004.pdf]

1. Age
2. Gender
    a. Man
    b. Woman
3. Do you live
    a. Alone
    b. With someone
4. Does your household include a dog?
    a. Yes
    b. No.
5. Do you have children under 18?
    a. Yes
    b. No.
6. Are you a competitive athlete?
    a. Yes
    b. No.
7. On average, how much have you had recreational physical activity in the week before COVID time (Before March 2020)?

Intensity	Amount per week, MET units				
(Mark all four options)	None	<½ hour	1 hour	2 – 3 hours	≥ 4 hours
Normal walking or respective					
Brisk walking or respective					
Light jogging or respective					
Brisk jogging or respective					

8. On average, how much have you had recreational physical activity in the week during COVID time (After March 2020)?

Intensity	Amount per week, MET units				
(Mark all four options)	None	<½ hour	1 hour	2 – 3 hours	≥ 4 hours
Normal walking or respective					
Brisk walking or respective					
Light jogging or respective					
Brisk jogging or respective					

9. During the COVID period (after March 2020), have you had any injuries or illnesses that have affected the amount of physical exercise?
    a. Yes
    b. No
10. Alcohol use before COVID time (March 2020)
    a. On average, how many times a week have you consumed alcohol before COVID time?
    b. On average, how many doses of alcohol at a time have you used before Korona time? (One serving of alcohol is, for example, a bottle of medium beer, 12 cl of wine or 4 cl of strong liqueur)
11. Alcohol use during the COVID period (after March 2020)
    a. On average, how many times a week have you consumed alcohol during COVID?
    b. On average, how many doses of alcohol have you consumed at one time during Korona? (One serving of alcohol is, for example, a bottle of medium beer, 12 cl of wine or 4 cl of strong liqueur)
